# Climate change impacts on the global potential distribution of the human flea, *Pulex irritans,* and the global health risks

**DOI:** 10.1038/s41598-026-36420-6

**Published:** 2026-02-10

**Authors:** Hadeer Magdy, Magdi G. Shehata, Mona G. Shaalan, Eslam M. Hosni, Sara A. Al-Ashaal

**Affiliations:** 1 Research Lab of Biogeography and Wildlife Parasitology, Department of Entomology, Faculty of Science, Ain Shams University, Cairo, Egypt; 2 Department of Entomology, Faculty of Science, Ain Shams University, Cairo, Egypt

**Keywords:** *Pulex irritans*, MaxEnt, GIS, Vector-borne diseases

## Abstract

The human flea, *Pulex irritans*, is a hematophagous ectoparasite and medically significant vector of zoonotic pathogens, such as *Yersinia pestis* (plague), *Bartonella quintana* (trench fever), and *Rickettsia felis* (flea-borne spotted fever). Despite the public health significance of *P. irritans*, the potential impacts of climate change on its global distribution were unstudied before. In this study, we created an ecological niche model (ENM) through integrating 564 georeferenced records and 15 bioclimatic variables using the Maximum Entropy (MaxEnt) algorithm to project the current and future habitat suitability of *P. irritans* under two high-emission scenarios (SSP370 and SSP585) for 2050 and 2070 from three General Circulation Models (GCMs). DIVA- GIS was used to confirm the current predictions. Results revealed that the Model’s performance was robust with high predictive accuracy (AUC = 0.898; TSS = 0.6), identifying annual mean temperature (Bio1) with 55.9% contribution as the primary distribution variable. The models project that many species across North and South America, Europe, Asia, Australia, and Africa will expand their ranges toward higher latitudes. Regions once deemed unsuitable, including northern Europe, Canada, and Russia, are forecast to become suitable habitats as these species shift their geographical distribution. On the other hand, habitat loss was predicted in Africa and Australia due to extreme warming. Two-dimensional niche analysis revealed the broad tolerances of *P. irritans* (2–25 °C; 0–2200 mm), confirming its invasive potential. These shifts correlate with increased plague risk in temperate zones, as warmer temperatures accelerate flea life cycles and pathogen transmission efficiency. Our findings provide the first global assessment of climate-driven redistribution *of P. irritans,* highlighting the urgent need for surveillance in vulnerable regions to mitigate emerging vector-borne disease threats.

**Supplementary Information:**

The online version contains supplementary material available at 10.1038/s41598-026-36420-6.

## Introduction

Vector-borne diseases (VBDs) account for more than 17% of global infectious diseases, primarily transmitted by arthropods, including mosquitoes, ticks, and fleas^1^. While fleas receive less public health attention than mosquitoes or ticks, they remain critical vectors of zoonotic pathogens^2^, with broad geographic distributions highlighting their medical and veterinary significance^3^.

Within the family Pulicidae (Siphonaptera), which exhibit diverse host-specificity patterns and ecological adaptations, contributing to their global prevalence^4^, *Pulex irritans* L. 1758 (human flea) is the most extensively studied species^3^. The human flea is a wingless, blood-feeding ectoparasite that mainly targets humans but can also infest other mammals, such as pets and wild animals^3^. This cosmopolitan vector transmits *Yersinia pestis* (plague), *Rickettsia typhi* (murine typhus), and is associated with flea-borne spotted rickettsiosis^2^. Morphologically, *P. irritans* lacks genal and pronotal ctenidia^5^. Recent decades have seen expanded geographic ranges of zoonotic VBDs, partly due to climate change^6^.

Climate change is altering global weather patterns and elevating temperatures^7^. The World Health Organization (WHO) projects climate-related factors will cause 250,000 additional annual deaths from 2030 to 2050, driven by malnutrition, heat stress, and infectious diseases^8^. Notably, VBD distributions are shifting as temperature and precipitation changes affect vector ecology, including reproduction rates and pathogen transmission efficiency^6^. Fleas exhibit particular sensitivity to climatic variables; minor temperature fluctuations can alter their life cycles, geographic ranges, and pathogen incubation periods^2^. Consequently, temperate regions face growing risks of flea-borne epidemics as warming enables vector colonization^9^, while extreme weather events may further disrupt ecological balances^10^. Unlike host-restricted vectors, fleas display ecological plasticity, allowing them to thrive in diverse environments where suitable microclimatic conditions exist^11^.

Geospatial tools such as Geographic Information Systems (GIS) and ecological niche modeling (ENM) are critical for predicting these risks^12^. MaxEnt is recognized for its exceptional predictive accuracy in species distribution modelling^13^, maintaining reliable performance even when working with limited occurrence data. This powerful analytical tool combines known species distribution records with environmental variables to model ecological preferences, then extrapolates these findings across various geographic regions and future climate scenarios^14^. Among ecological niche modelling approaches, MaxEnt demonstrates superior predictive performance^15^. Its unique capacity to forecast future habitat suitability, a capability absent in many alternative models, made it the ideal choice for our investigation. It is widely recognized in ecological studies for analyzing species distributions based on climatic variables^13^^,^^16^. This study will model the current global distribution of *P. irritans* and project its future habitat suitability (2050–2070) under high-emission scenarios (SSP370, SSP585) using three General Circulation Models to identify high-risk regions vulnerable to invasion, evaluate the expansion potential of *Pulex irritans*, and produce actionable maps to guide targeted surveillance and mitigation efforts against this vector.

## Materials and methods

### Occurrence data

Occurrence records of *Pulex irritans* were downloaded from the Global Biodiversity Information Facility (GBIF.org; accessed on 3 January 2022). Most records were clustered in North America. To minimize sampling bias in ecological niche modeling (ENM), we applied a three-step filtering protocol^16^: (1) records without precise geographic coordinates were excluded, (2) duplicate entries were removed, and (3) spatial rarefaction was performed using the SDM Toolbox in ArcGIS v10.3 (Universal Tools—Spatially Rarefy Occurrence Data) to mitigate overrepresentation. After filtering, 564 high-quality records remained and were converted to CSV format for modeling the current and future global distribution of the human flea.

### Climatic data

Historical and future climate data were obtained from WorldClim version 2.1 (www.worldclim.org) at a 2.5 arc-minute resolution (~ 5 km)^17^ We initially considered 19 bioclimatic variables (derived from 1970 to 2000 monthly temperature and precipitation records) but excluded four (Bio8, Bio9, Bio18, and Bio19) due to known spatial artifacts^16^. The remaining 15 variables were converted to ASCII format using ArcGISv10.3.

To evaluate climate change impacts, we employed three General Circulation Models (GCMs): BCC-CSM2-MR (China), IPSL-CM6A-LR (France), and MRI-ESM2-0 (Japan) under two high-emission scenarios (SSP370 and SSP585). Projections were generated for 2050 (2041–2060 average) and 2070 (2061–2080 average). All datasets were converted to ASCII format for analysis. We analyzed 12 model combinations (3 GCMs × 2 SSPs × 2 time periods). The mean predicted distribution for each SSP across all GCMs was computed for 2050 and 2070 and compared with the current distribution.

### Modeling approach

**Fig. 1:**
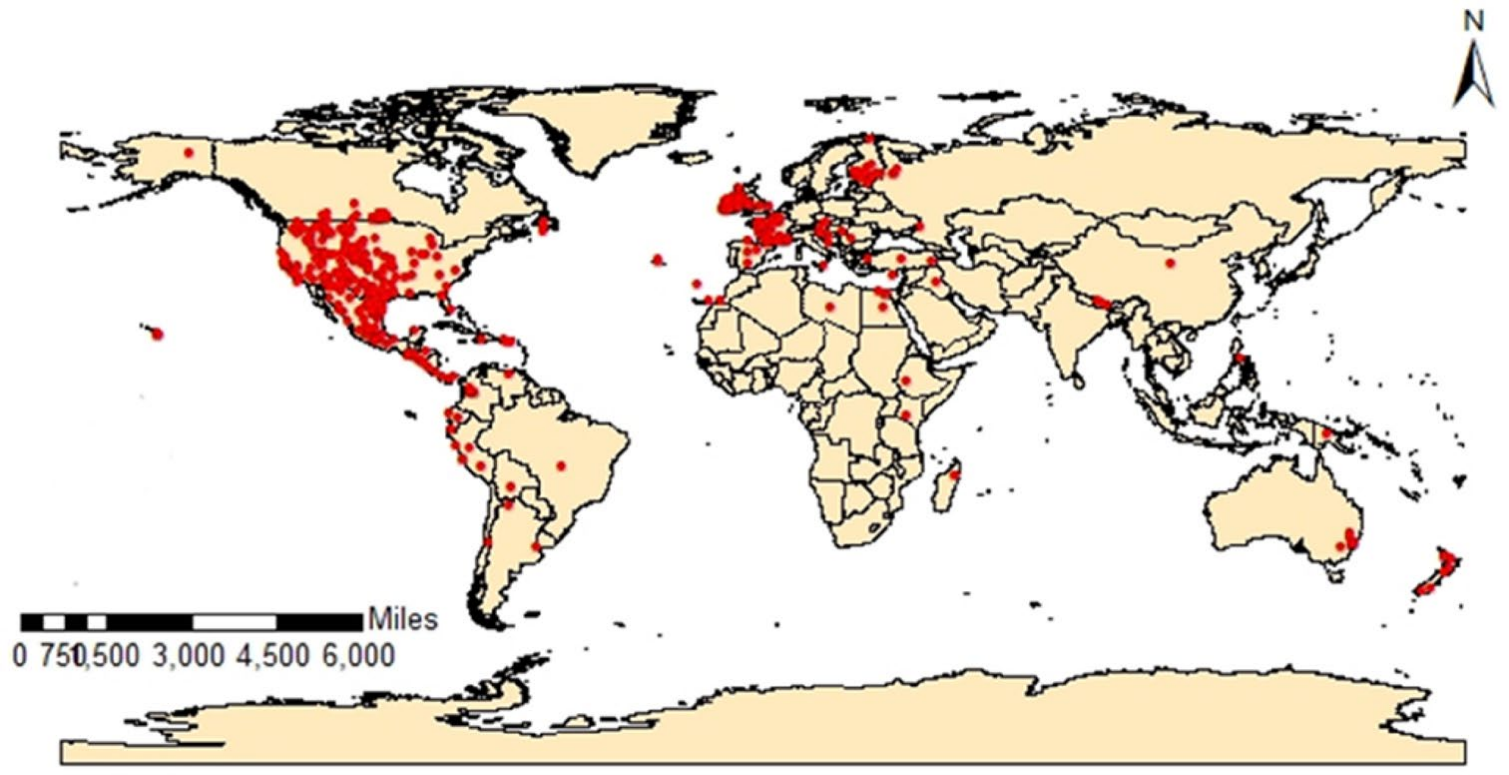
The occurrence records of *Pulex irritans* used for the species distribution model (SDM).

We performed species distribution modeling using MaxEnt (version 3.4.1), selected for its capacity to integrate non-climatic variables (e.g., land cover, distance) and its maximum entropy algorithm. Occurrence records were randomly partitioned into 75% training and 25% testing datasets^18^. The model employed 10,000 background points, 1,000 maximum iterations, and tenfold cross-validation to ensure robustness. Habitat suitability maps were categorized into six classes (Unsuitable, Low, Medium, High, Very High, Excellent) using the Natural Breaks (Jenks) method in ArcGIS v10.3 (Fig. 1).

### Model evaluation

The performance of the models was evaluated using two metrics: the Area Under the Curve (AUC) of the Receiver Operating Characteristic (ROC) and the True Skill Statistic (TSS). The AUC score ranges from 0 (indicating random prediction) to 1 (indicating perfect discrimination), with values above 0.75 considered well-fitting and those below 0.5 considered poor-fitting. Additionally, the TSS was applied to further assess model accuracy, with scores ranging from 0 to 1. A TSS value close to 1 suggests a strong agreement between the model predictions and the actual species distribution, while a value near 0 indicates weak predictive performance^19^.

### Two-dimensional niche analysis and limiting factors

An investigation into the two-dimensional ecological niche of the human flea was conducted using the DIVA-GIS software platform^20^. The Envelope Test was applied to model the tolerance range of species and assess habitat suitability based on two key bioclimatic variables: annual mean temperature (Bio1) and annual precipitation (Bio12). Furthermore, a limitation factor map was generated using the bioclimatic statistical modeling tool within DIVA-GIS. This spatial analysis identified and visualized the specific climatic variables most influential in limiting the geographical distribution of *P. irritans*.

## Result

### Model evaluation of bioclimatic variables

We employed the Area Under the Curve (AUC) and the True Skill Statistic (TSS) to assess the precision and efficacy of our MaxEnt model. Most maximum entropy models use the area under the curve (AUC) as a crucial statistic; higher values show that the model is doing better. Supplementary Figure S1 showed that our model had a significant impact on predicting the environmental adaptability of the human flea, as it attained an AUC of 0.898 (Fig. S1). Applying the TSS further validated the reliability of the model; it produced a score of 0.6, indicating strong predictive quality. The values of TSS greater than 0.5 are generally regarded as satisfactory.

**Fig. 2:**
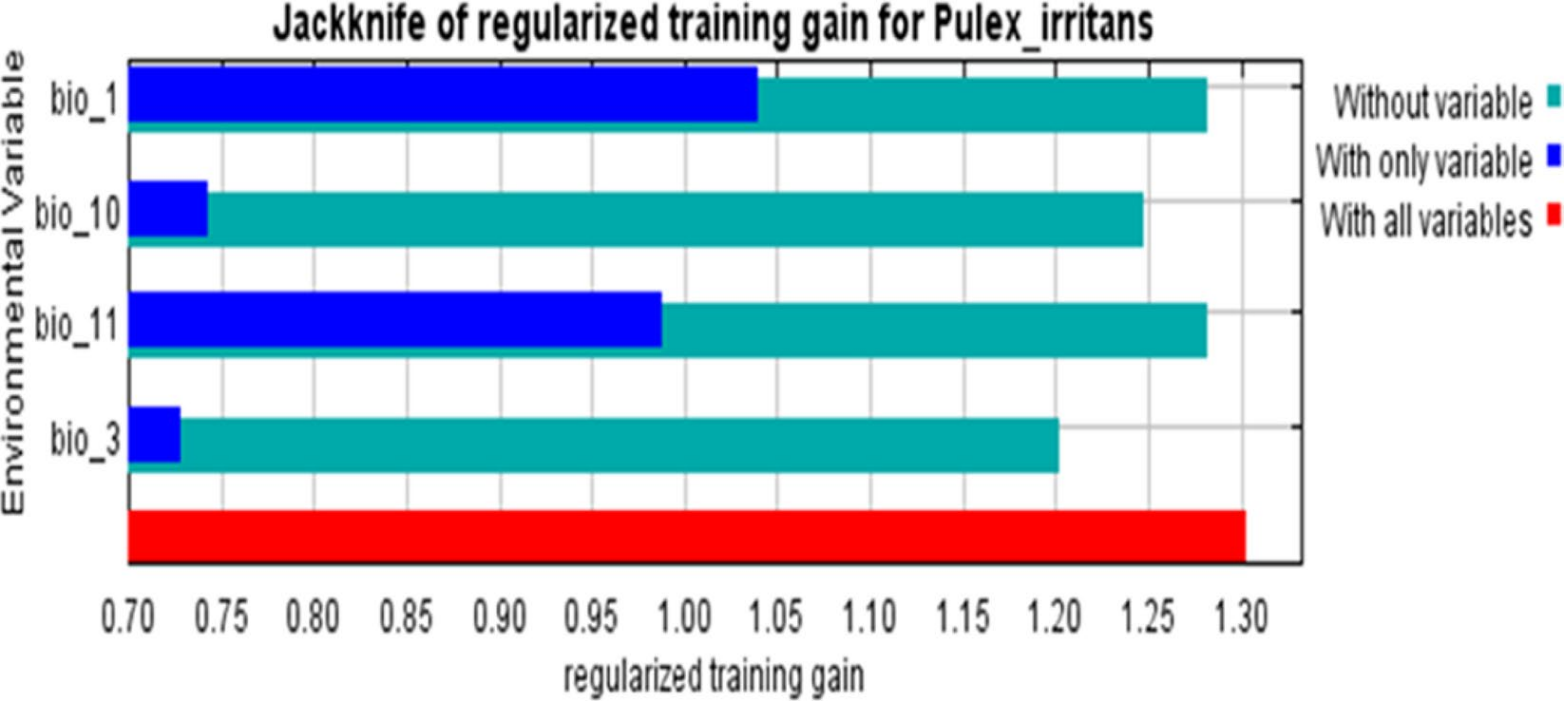
The Jackknife test for *Pulex irritans* showing the most effective environmental variables.

The results of the Jackknife test (Fig. 2 and Table 1) showed contributions of the bioclimatic variables to the prediction model. The results demonstrated that when trying to predict the dispersion of *P. irritans*, temperature-related variables are crucial. Annual mean temperature (Bio1) was the most influential variable with 55.9% of the model’s predictive power, along with isothermality (17.3%). The next most important factor was the mean temperature of the coldest quarter (Bio11), and lastly, the mean temperature of the warmest quarter (Bio10) was 12.2%. In addition, the response curves showed that *P. irritans* thrived in an ideal annual mean temperature range of 10–20 °C (Fig. S2).

**Table 1:** Relative percentages of bioclimatic variables used in MaxEnt to model the current and future habitat suitability of *Pulex irritans.*

Bioclimatic variables	Description	Contribution percentage (%)
Bio 1	Annual mean temperature	55.9
Bio 3	Isothermality (bio2 / bio7) (*100)	17.3
Bio 11	Mean temperature of coldest quarter	14.7
Bio 10	Mean temperature of warmest quarter	12.2

### Current potential distribution of *Pulex irritans*

**Fig. 3:**
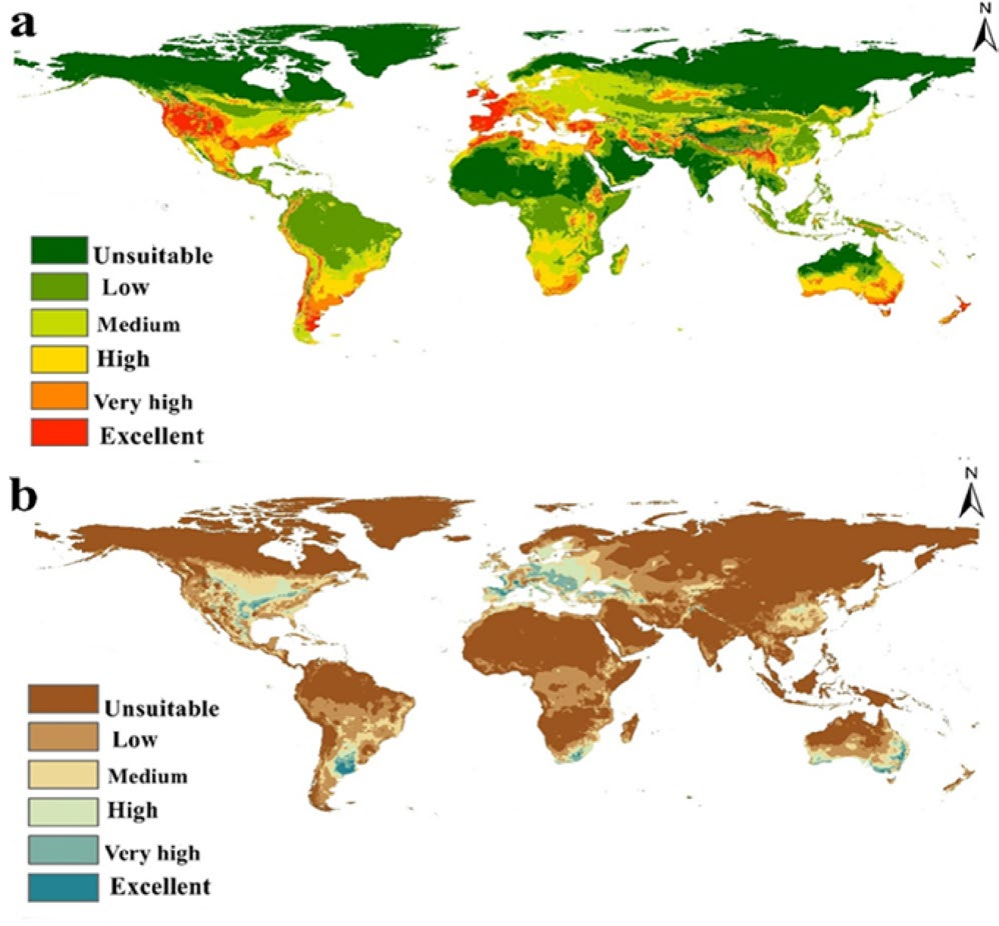
Current potential distribution of *Pulex irritans* (**a**) Using MaxEnt v. 3.4.1 and ArcGIS v. 10.3, (**b**) Using DIVA-GIS.

Based on the existing climate circumstances, *Pulex irritans* could potentially be distributed worldwide. Both the MaxEnt (Fig. 3a) and DIVA-GIS (Fig. 3b) models demonstrated strong agreement in predicting the current habitat suitability, which closely matches the documented distribution of this species (Fig. 1). The distribution in our model divided into regions of excellent, high and very high suitability such as western and southern united states, western coast of South America especially Peru and Chile. As well as Uruguay and Argentina, which lie in the southern region of South America. The old-world continents (Asia, Africa and Europe) generally exhibited low to medium distribution except for some highly suitable regions such as the coastal region of most countries that overlook the Mediterranean Sea from Africa (Egypt, Libya, Algeria) in addition to Tunisia and Morocco, from the Asian side, Turkey, Syria, Jordan and Lebanon in addition to Spain, Portugal, France, Germany, Italy, Greece and Cyprus from the European side demonstrated highly suitable regions for *P. irritans*. Furthermore, Ireland and the United Kingdom showed a high distribution of this species. Southern countries of Africa, some parts of Iran, Pakistan, and Afghanistan, in addition to parts of South China, showed high suitability for the human flea distribution. The majority of Australia, especially the eastern Southern coast and New Zealand, are highly suitable regions for *P. irritans*. On the other hand, the model showed low and unsuitable regions such as Russia, northern Europe, Canada, middle Africa, India, and northern Australia.

### Future potential distribution (2050 and 2070)

**Fig. 4:**
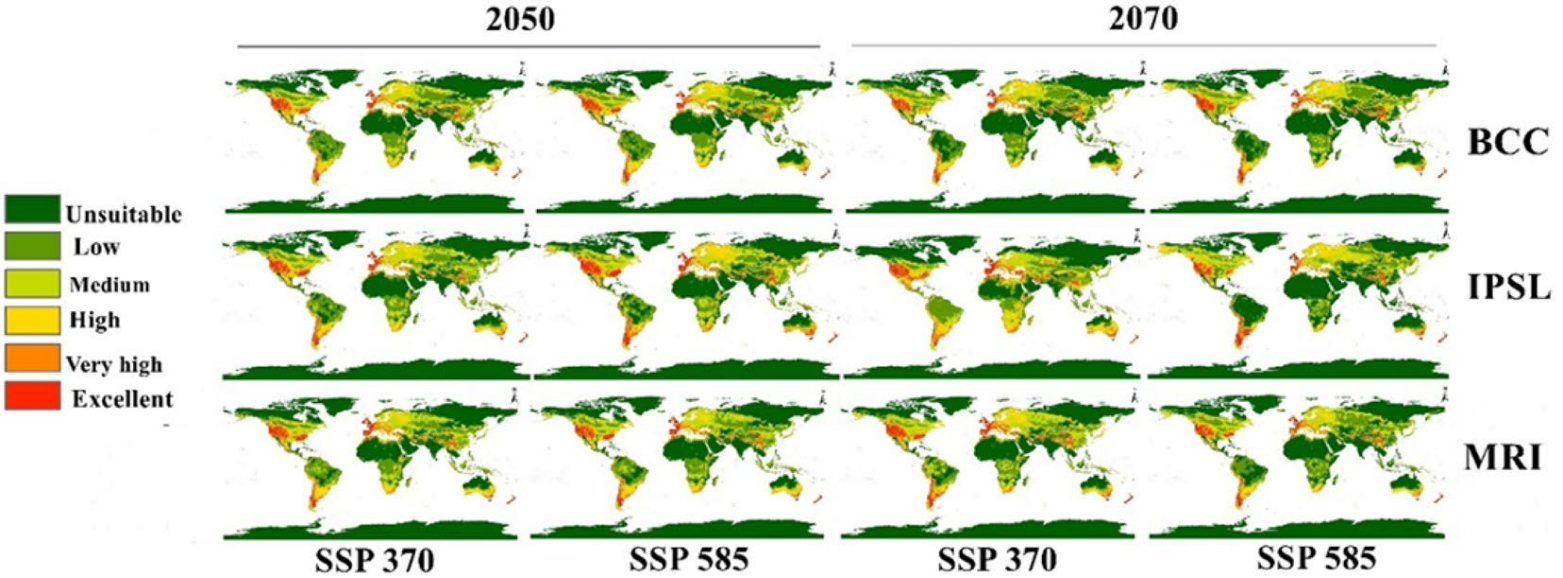
Predicted future distribution of *Pulex irritans* under the SSP 370 and 585 for three GCMs (MaxEnt v. 3.4.1 and ArcGIS v. 10.3).

**Fig. 5:**
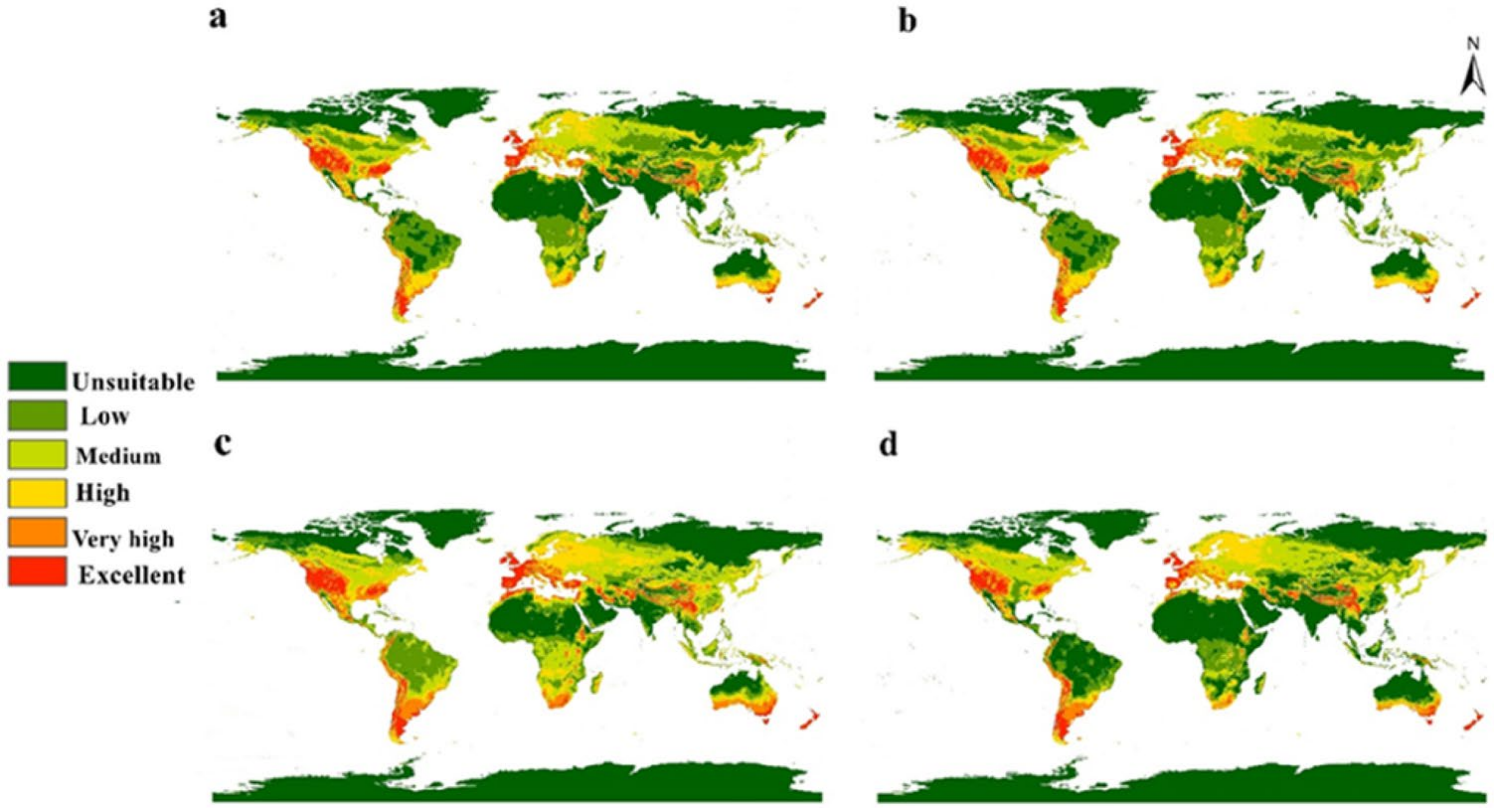
Predicted maps for the mean of three future GCMs using four SSP scenarios: (**a**) SSP 370 for 2050; (**b**) SSP 585 for 2050; (**c**) SSP 370 for 2070; and (**d**) SSP 585 for 2070.

**Fig. 6:**
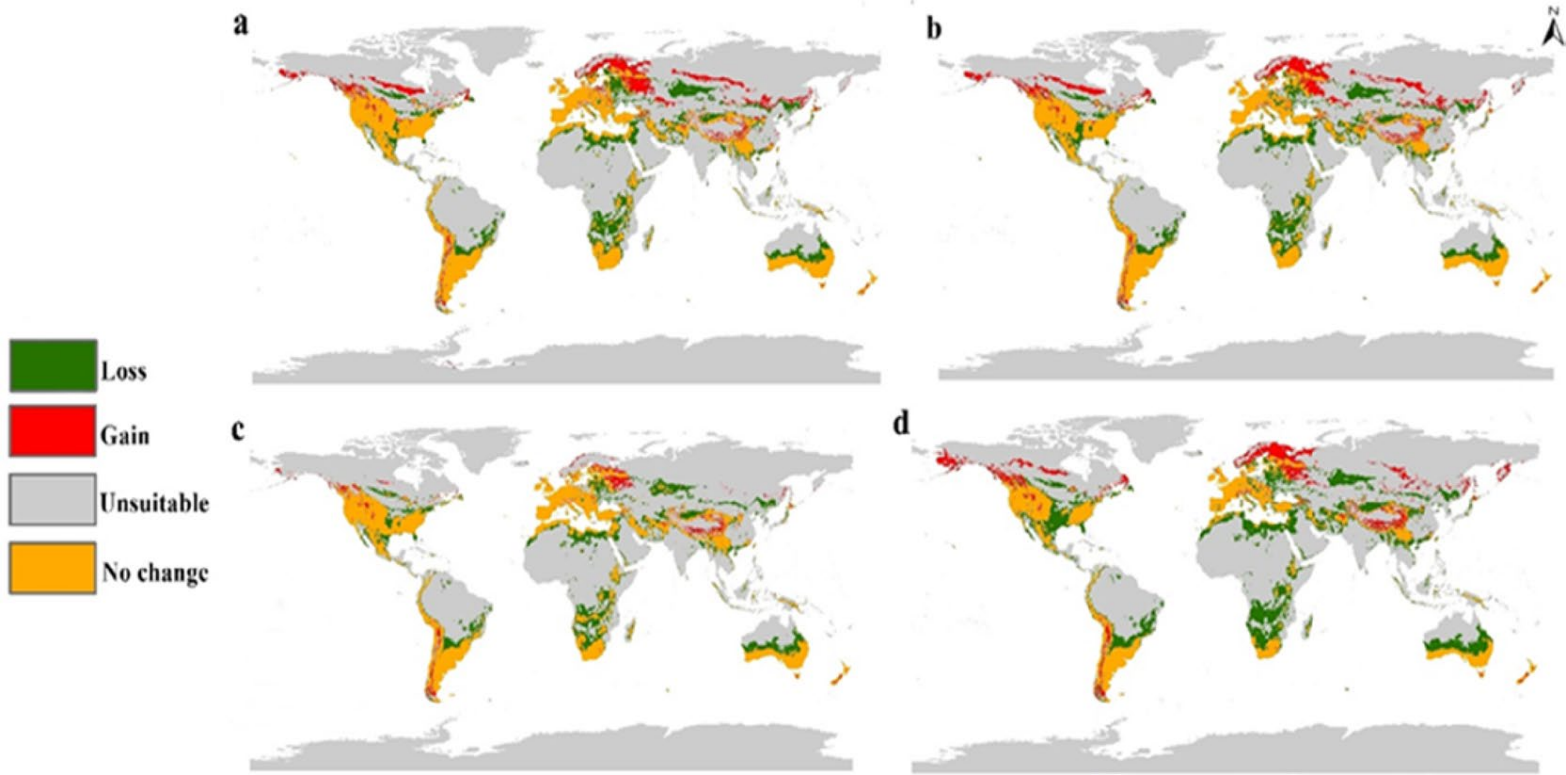
Calibration maps showing gain and loss in habitat suitability of *Pulex irritans* through the four mean future scenarios against the current status: (**a**) SSP 370 for 2050; (**b**) SSP 585 for 2050; (**c**) SSP 370 for 2070; and (**d**) SSP 585 for 2070.

The future status of the human flea during 2050 and 2070 under two high emission scenarios (SSP 370 and 585) was evaluated using three GCMs (Fig. 4). The average of these models was computed to summarize the projected shifts in the habitat suitability of *P. irritans* (Fig. 5). Furthermore, we quantified suitability loss and gain to evaluate variations between current and future conditions (Fig. 6). The future distribution model from 2050, SSP 370 until 2070, SSP 585 showed clear northern shift in the habitat suitability of *P. irritans* toward the northern hemisphere, this shifting includes Canada, northern Europe and Russia which are unsuitable regions in the current distribution. The prominent gain of new suitable niches is met by a clearly visible loss in habitat suitability, especially in Africa and Australia.

### Two-dimensional niche analysis and limitation factor map

**Fig. 7:**
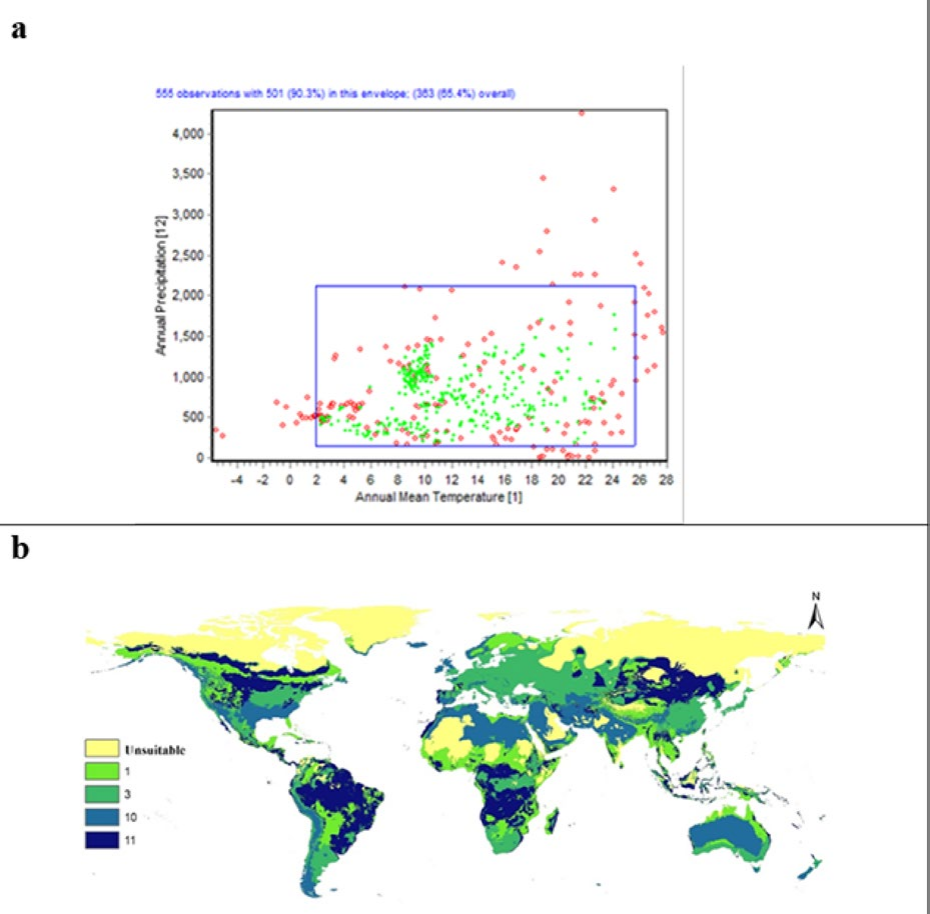
DIVA-GIS analysis of climatological variables and their effects on *Pulex irritans:* (**a**) Two-dimensional niche between annual mean temperature (Bio1) and annual precipitation (Bio12), (**b**) Limitation factor map of the selected variables.

The two-dimensional niche of the human flea was modeled using the envelope test, through analyzing annual mean temperature (Bio1) and annual precipitation (Bio12) (Fig. 7a). The envelope test (Fig. 7a) showed *P. irritans* tolerates wide range of annual mean temperatures (2–25 °C) and annual precipitation of 0–2200 mm, indicating broad ecological adaptability and tolerance of this species across different climatological conditions. However, some ecological variables limit and control this broad adaptability, such as the mean temperature of the coldest quarter in Asia and South America, and the mean temperature of the warmest quarter in Australia and the Middle East, as shown in the limitation factor map (Fig. 7b).

## Discussion

Global climate change, characterized predominantly by rising temperatures since the twentieth century, poses significant challenges to vector-borne disease management. The Sixth Assessment Report (2021) of the Intergovernmental Panel on Climate Change (IPCC) confirms that global temperatures have already increased by approximately 1°C since 1850–1900, with projections indicating a likely reach or exceedance of 1.5°C within the next two decades⁷. These climatic shifts present critical implications for the spatial ecology of disease vectors, including *P. irritans*, necessitating a comprehensive assessment of their potential redistributions.

The intersection between climate-driven vector redistribution and sustainable development represents a mounting global concern. Climate Action (SDG 13) and the goal to end epidemics of diseases (SDG 3.3) are fundamentally interconnected through vector-borne disease dynamics. As global temperatures rise and precipitation patterns shift, the habitat suitability for medically significant vectors such as *P. irritans* is projected to expand into previously unaffected temperate zones and high-altitude regions^21^. This geographical expansion threatens substantial progress toward disease elimination goals, given that *P. irritans* serves as a competent vector for multiple zoonotic pathogens, including *Yersinia pestis* (plague), Rickettsia typhi (murine typhus), and *Bartonella* spp. (bartonellosis), all of which contribute significantly to morbidity and mortality in vulnerable populations^2^.

Our modeling approach integrates Geographic Information Systems (GIS) with Maximum Entropy (MaxEnt) algorithms to provide a robust predictive framework for assessing species distributions under changing climatic conditions^22^. This methodology enables quantification of distributional shifts in response to temperature increases, altered precipitation patterns, and other bioclimatic factors. The predictive capability of this integrated approach proves essential for evaluating invasion risk and identifying emerging high-risk zones, thereby informing evidence-based, preemptive mitigation strategies^23^. The superiority of MaxEnt among niche modeling approaches has been well-documented, with Elith et al.^15^ demonstrating that MaxEnt outperformed 15 alternative models in predictive accuracy.

The strong predictive performance of our model, evidenced by high AUC (0.898) and TSS (0.6) values, demonstrates robust alignment between projected habitat suitability and documented occurrence patterns of *P. irritans*. The dominance of temperature-related variables in shaping species distribution, as revealed through Jackknife analysis, aligns with previous findings for congeneric species. Notably, Wang et al.^24^ reported similar temperature dependence in *Pulex simulans*, reinforcing the thermal sensitivity characteristic of the genus. Temperature serves as a fundamental determinant of flea biology, directly influencing developmental rates across larval and pupal stages^25^, while simultaneously modulating pathogen transmission efficiency through effects on vector competence and plague bacillus multiplication within the flea gut^26^^,^^27^.

Our current distribution projections demonstrate strong concordance with existing occurrence data and exhibit substantial overlap with suitable areas identified for *P. simulans* under contemporary climate conditions^24^. The identification of the 100th meridian as a significant biogeographic boundary in North America, with western states exhibiting superior environmental suitability compared to eastern regions, corresponds remarkably with the heterogeneous distribution of plague across the United States^28^. This spatial pattern reflects the enzootic persistence of plague in western wildlife populations while remaining largely absent east of this meridian. The model’s accuracy in capturing this biogeographic divide underscores the robust integration of occurrence records with ecological constraints, generating reliable realized niche projections.

The Mediterranean Basin emerges as a particularly critical region for *P. irritans* distribution, with high suitability spanning African, Asian, and European coastlines. This finding carries substantial epidemiological significance given the region’s extensive network of ports and trade routes, which historically facilitated plague dissemination and continue to represent potential pathways for vector dispersal. The role of maritime trade routes in pathogen spread has been extensively documented, with Schaub & Vogel^29^ demonstrating that the first two plague pandemics originated in Central Asian steppes and spread via the Silk Road to Europe, while the third pandemic was globally disseminated through steamship networks connecting major port cities. The contemporary suitability of Mediterranean coastal areas necessitates enhanced surveillance and quarantine measures at ports to prevent inadvertent vector translocation.

Future projections across multiple GCMs under high-emission scenarios (SSPs 370 and 585) reveal substantial poleward range expansions, with northern European nations including Finland, Sweden, and Norway, alongside regions of Russia and China, projected to experience high to very high suitability by 2050–2070. Throughout the Americas, Canada, the northern United States, and southern South American nations face similar prospects. These projections align with broader predictions by Medlock & Leach^21^, who indicated that global warming would enable ectoparasites like *P. irritans* to colonize higher latitudes and elevations. Notably, our findings regarding future *P. irritans* distributions show strong spatial concordance with the contemporary ranges of *Peromyscopsylla hesperomys* and *Orchopeas sexdentatus*, recognized plague vectors in North America^30^, suggesting potential for similar ecological roles in newly suitable habitats.

Conversely, tropical and subtropical regions, particularly across Africa and Australia, are projected to experience habitat loss due to temperature increases exceeding species tolerance thresholds. This spatial heterogeneity in climate change impacts simultaneous range expansion in temperate zones and contraction in currently suitable tropical areas underscores the complex nature of climate-driven distributional shifts. Such patterns have been documented across diverse taxa responding to warming conditions and represent a fundamental challenge for vector management strategies.

Our modeling framework, while robust, acknowledges inherent limitations. The exclusive reliance on climatic predictors, though supported by previous research^31–33^, necessarily simplifies the complex ecological factors governing species distributions. Integration of additional variables, including human population density, land cover patterns, and host species distributions, would likely enhance model precision. However, the absence of reliable projections for these variables under future climate scenarios currently limits their incorporation into predictive frameworks. Future research should prioritize the development of comprehensive models integrating environmental, anthropogenic, and biological factors, alongside the formulation of targeted intervention strategies for high-risk regions. Such advances are essential for proactive management of both the ecological and public health consequences of *P. irritans* range expansion in a warming world.

The implications of our findings extend beyond academic interest to practical public health preparedness. Regions projected to gain habitat suitability, particularly those at higher latitudes without recent plague endemicity, may lack established surveillance infrastructure and public health capacity to detect and respond to novel vector-borne disease threats. Conversely, areas experiencing habitat loss may face altered disease dynamics as vectors and reservoirs redistribute spatially. Both scenarios necessitate adaptive management approaches informed by continuous monitoring and updated predictive modeling as climate trajectories and species responses become clearer.

## Conclusion

We have modeled the potential effects of ongoing climate change on the global distribution of suitable habitats for *Pulex irritans* across multiple future climate scenarios and socioeconomic pathways. Our projections indicate a significant expansion in the geographic range of *P. irritans*. Maximum entropy (MaxEnt) analysis, applied across 12 future scenarios, confirms that this species will pose a substantial public health threat in both the near (2021–2040) and long-term (2081–2100) future. Climate-driven shifts in environmental suitability are expected to alter the availability of favorable habitats, influencing the species distribution and establishment potential. Temperatures emerged as the key driver of distribution shifts. Notably, our models predict an increase in highly suitable habitats for *P. irritans*, elevating the risk of vector-borne pathogen transmission in previously non-endemic regions. However, these effects are spatially heterogeneous; the effects are not limited to range expansion, while some historically suitable areas may see increased environmental suitability, others could become less hospitable due to shifting climatic conditions.

## Supplementary Information

Supplementary Information.

## Data Availability

The datasets generated and/or analyzed during the current study are available from the corresponding author on reasonable request.
