# Supplementary material for: Climate change impacts on the global potential distribution of the human flea, Pulex irritans, and the global health risks
Source: Sci Rep. 2026 Feb 10;16:5944. doi: 10.1038/s41598-026-36420-6 (PMC12895019; doi:10.1038/s41598-026-36420-6)
Supplement: Supplementary file 1 — Supplementary Information. [file 41598_2026_36420_MOESM1_ESM.docx]

Figure S1. Receiver operating characteristic (ROC) curve for the data showing AUC value.


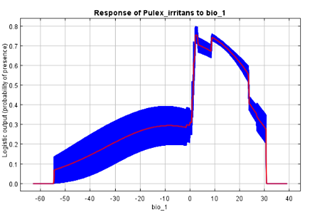


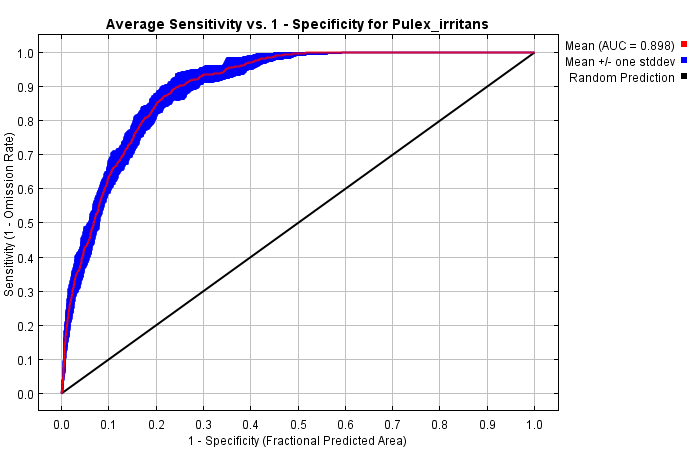


Figure S2. The response curve of the most effective environmental factors (Bio1) affecting the distribution of *Pulex irritans*; the shown values are the average of ten replicate runs.
